# Sleep duration and all-cause mortality in the elderly in China: a population-based cohort study

**DOI:** 10.1186/s12877-020-01962-5

**Published:** 2020-12-30

**Authors:** Yanfeng Ren, Maohua Miao, Wei Yuan, Jiangwei Sun

**Affiliations:** 1 Department of Health Statistics, School of Public Health, Weifang Medical University, Shandong, China; 2 NHC Key Lab. of Reproduction Regulation, Shanghai Institute of Planned Parenthood Research), Fudan University, Shanghai, China; 3 Institute of Environmental Medicine, Karolinska Institutet, Stockholm, Sweden

**Keywords:** Sleep duration, All-cause mortality, Elderly, Cohort

## Abstract

**Background:**

Although a U-shaped association between sleep duration and all-cause mortality has been found in general population, its association in the elderly adults, especially in the oldest-old, is rarely explored.

**Methods:**

In present cohort study, we prospectively explore the association between sleep duration and all-cause mortality among 15,092 participants enrolled in the Chinese Longitudinal Healthy Longevity Survey (CLHLS) from 2005 to 2019. Sleep duration and death information was collected by using structured questionnaires. Cox regression model with sleep duration as a time-varying exposure was performed to calculate the hazard ratios (HRs) and 95% confidence intervals (CIs). The dose-response association between them was explored via a restricted cubic spline function.

**Results:**

During an average follow-up of 4.51 (standard deviation, SD: 3.62) years, 10,768 participants died during the follow-up period. The mean (SD) age of the participants was 89.26 (11.56) years old. Compared to individuals with moderate sleep duration (7–8 hours), individuals with long sleep duration (> 8 hours) had a significantly higher risk of all-cause mortality (HR: 1.13, 95%CI: 1.09–1.18), but not among individuals with short sleep duration (≤ 6 hours) (HR: 1.02, 95%CI: 0.96–1.09). Similar results were observed in subgroup analyses based on age and gender. In the dose-response analysis, a J-shaped association was observed.

**Conclusions:**

Sleep duration was associated with all-cause mortality in a J-shaped pattern in the elderly population in China.

## Background

As one of fundamental components of daily life, sleep is crucial for maintaining normal physiological function. According to the consensus of the National Sleep Foundation in the United States, the recommended level of sleep duration for young and older adults is 7–9 and 7–8 hours, respectively [1]. Inappropriate sleep duration, as reported by numerous studies, was associated with various adverse health outcomes including obesity, type 2 diabetes, cardiovascular disease, cancer, cognitive decline as well as total and cause-specific mortality [2–6]. A U-shaped pattern of the effects has been observed, that is long and short sleep duration increased the risk of all-cause mortality in general population [7, 8].

As aging is a big challenge faced by countries due to the advances in medical technology, improvements in lifestyle, and socioeconomic development, exploring how sleep duration is associated with all-cause mortality among the elderly population become more urgent. Compared with young adults, the elderly population are more likely to be affected by many factors, such as unhealthy lifestyles, menopausal status, chronic disease and mental health [9, 10], thus the relationship of sleep duration with all-cause mortality may be more complex. In addition, several issues have not been fully addressed in previous studies. First, the oldest-old population, such as octogenarians (person with 80–89 years old), nonagenarians (person with 90–99 years old), or centenarians (person with more than 100 years old), was rarely enrolled, which makes a challenge to explore the effect of sleep duration on all-cause mortality among those specific population. Second, sleep duration was usually transformed to categorical variable in previous studies, thus the dose-response relationship between them have not been investigated in detail. Third, previous studies were more likely to only evaluate the effect of sleep duration that measured at baseline [5, 11], while failing to accounting for its variety after enrollment would translate into exposure misclassification and cause immoral time bias.

In present study, to fill the aforementioned knowledge gap, we used data from the Chinese Longitudinal Healthy Longevity Survey (CLHLS) to explore the relationship between sleep duration and all-cause mortality in the elderly adults, especially in the oldest-old.

## Methods

### Study design

**Fig. 1:**
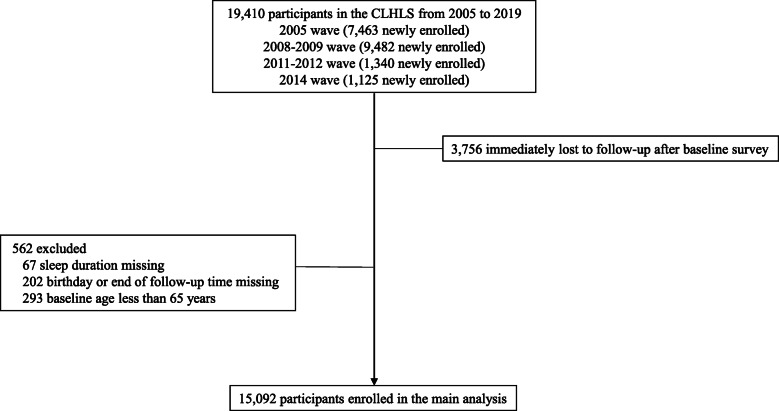
Flow chart of the selection of participants, a cohort study of the elderly adults in China, 2008–2019

We performed a cohort study by using data from the Chinese Longitudinal Healthy Longevity Survey (CLHLS). A detailed information of CLHLS has been previously published [12]. Briefly, CLHLS, aimed to understand the factors influencing the health of the elderly adults (aged 65 years or above), especially the oldest-old (aged 80 years or above), is a nationwide survey conducted in 22 provinces in China, which covers about 85 percent of the total population [13]. A targeted and disproportionate sampling method was adopted to obtain a sample with enough sufficient number of the oldest-old. Eight waves were conducted in 1998, 2000, 2002, 2005, 2008–2009, 2011–2012, 2014 and 2018–2019, respectively, and sleep duration was initially measured from 2005 wave. Due to lacking of follow-up information, participants enrolled in 2018–2019 wave were excluded. A total of 19,410 participants were initially enrolled in present study, and among them, 3,756 participants only having baseline information and 562 participants lacking information in sleep duration, birthday or end of follow up time, or younger than 65 years at baseline were further excluded from the analysis. Eventually, 15,092 participants were included. The flow chart of the participant selection was shown in Fig. 1.

### Sleep duration measurement and death assessment

Sleep duration was assessed by the question “how long do you usually sleep”. The participants or their close family members provided the information in the face to face interview. Participants were then categorized into 3 groups based on following criteria: short (≤ 6 h), moderate (7–8 h), or long (> 8 h) sleep duration.

Date of death was confirmed via family member or the village doctor. Cause-specific mortality, however, was not considered in present study due to two main reasons: a). many of the elderly individuals die in a natural way at home rather than in hospital, thus cause of death were not recorded; b). cause of death that reported by family member were imprecise and unreliable [14]. All participants were followed from enrollment until death, lost to follow-up, or 31 July 2019, whichever came first.

### Covariates assessment

To minimize the effect of potential confounders, socio-demographic information, lifestyle factors and health status of participant were adjusted. Socio-demographic information included age, sex (male/female), enrollment year (categorical variable: 2008, 2011, and 2014), province of residence (categorical), residence (city, town and rural area), ethic (Han/others), education (illiterate/primary school/middle school or above), marriage status (married/others), occupation (farmer or manual, clerical, professional, and others) and access to medical service (yes/no). Lifestyle factors included smoking (never, ever, and current smoker), drinking (never, ever, and current drinker), exercise (never, ever, and current exerciser), food diversity (measured by food diversity score), and social activity engagement (measured by social activity score). Health status information included activities in daily living (ADL, measured by ADL score), physical performance (measured by physical performance score), cognitive impairment (measured by Mini-Mental State Examination (MMSE) score) and chronic disease status (measured by chronic disease score). The definition of the abovementioned scores was described in one of our papers [15].

### Statistical analyses

Cox regression models with baseline age as the underlying time scale were used to explore the association between sleep duration and all-cause mortality. As sleep duration could change after enrollment, we, therefore, modeled sleep duration as a time-varying exposure in Cox regression models to minimize the misclassification of exposure and immoral time bias [16]. A participant, for example, contributed person-years to moderate group if he/she had a sleep duration of 7 to 8 hours, while contributed to the long group from the date of being recorded with a long sleep duration (> 8 h), until he/she was recorded with a different sleep status or he/she reached one of the above mentioned follow-up end points.

A structured scheme of confounder adjustment was used to test the robustness of our findings. We first included age (underlying time scale) and sex as covariates in model 1. Then we adjusted for enrollment year, province of residence, residence, ethic, marriage status, occupation, access to medical service, smoking, drinking, and exercise in model 2. In model 3, we further adjusted for ADL score (categorical: 6, 5, 3–4, and 0–2), physical performance score (categorical: 5, 2.5–4.5, and 0-2.5), MMSE score (categorical: 24–30, 18–23, and 0–17), food diversity score (categorical: 6–8, 4–5, and 0–3), social activity score (categorical: 5–8, 3–4, and 0–2), and chronic disease score (categorical: 0, 1–2, and ≥ 3). Subgroup analyses based on age and sex were performed, and whether heterogeneity existed within each subgroup were further explored by adding an interaction term of sleep status and either of the two factors in the Cox regression model. The proportional hazard assumption was evaluated via Schoenfeld residual plots and no violation was observed. Collinearity diagnostic was evaluated by the variance inflation factor and no collinearity among the covariates was found.

**Fig. 2:**
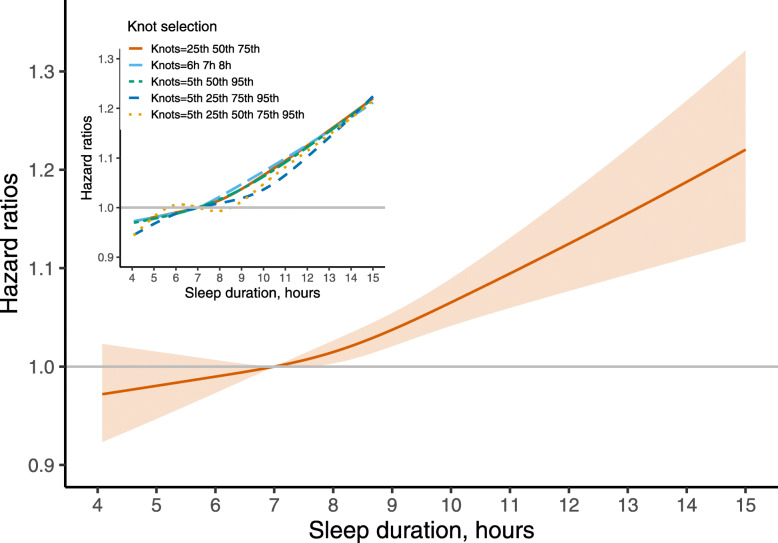
Adjusted dose-response association between sleep duration and risk of all-cause mortality among the elderly population in China, based on model 3. Sleep duration was modeled using a restricted cubic spline function with knots at 25th, 50th, and 75th percentiles of its distribution. The inset showed the HR of all-cause mortality risk based on different knots selection. The reference value was all set at 7 hours

To assess the dose-response association between sleep duration and all-cause mortality, sleep duration was modeled via a restricted cubic spline function with knots selected at 25th, 50th, and 75th percentiles of its distribution in the model 3. The reference value for sleep duration was chosen as 7 hours. We also explored whether the aforementioned knot selection was arbitrary by conducting sensitivity analyses using different knots selection, which was set at (6h, 7h, 8h), (5th, 50th, 95th ), (5th, 25th, 75th, 95th ), (5th, 25th, 50th, 75th, 95th ) of its distribution. We observed that compared to the results from the others, the result of selecting 25th, 50th, and 75th percentiles as knots was robust, as shown in Fig. 2.

Data analyses were performed using SAS version 9.4 (SAS Institute Inc, Cary, NC) and R platform. A two-sided *P* ≤ 0.05 was considered as statistically significant.

## Results

### Characteristics of the study population

During an average follow-up of 4.51 (standard deviation, SD: 3.62) years, 10,768 of the 15,092 participants died during the follow-up period. The mean (SD) age of the participants was 89.26 (11.56) years, ranging from 65 to 117, and 40.5% were male.

Baseline characteristics stratified by categories of sleep duration are shown in Table 1. Compared to the subjects with moderate sleep duration, those with short or long sleep duration were more likely to be older, female, married, farmer or manual, illiterate, and live in rural area, but less likely to be current smokers, drinkers, and exercisers. The proportion of the subjects with lower ADL, physical performances, MMSE, food diversity, and social activity scores in moderate sleep duration group were lower than that in short and long sleep duration group. The proportion of the subjects with lower chronic disease scores and access to medical service were lower in short sleep duration group but higher in long duration group. No significant difference was observed in ethic factor (Table 1).

**Table 1:** Baseline characteristics of the elderly adults, a cohort study in China, 2005-2019

Variables	Sleep duration			*P* value
	Short (≤ 6h), *n*=1,803	Moderate (7-8h), *n*=7,459	Long (> 8h), *n*=5,830	
Age at baseline, years				<0.0001
Mean ± SD	88.49 ± 11.12	87.07 ± 12.08	92.31 ± 10.26	
Categories, n(%)				<0.0001
<80 years	381 (21.13)	1999 (26.80)	693 (11.89)	
80-89 years	509 (28.23)	1888 (25.31)	1214 (20.82)	
90-99 years	522 (28.95)	2041 (27.36)	1939 (33.26)	
≥100 years	391 (21.69)	1531 (20.53)	1984 (34.03)	
Sex, n(%)				<0.0001
Male	662 (36.72)	3198 (42.87)	2255 (38.68)	
Female	1141 (63.28)	4261 (57.13)	3575 (61.32)	
Residence, n(%)				0.0035
City	328 (18.19)	1282 (17.19)	987 (16.93)	
Town	297 (16.47)	1383 (18.54)	959 (16.45)	
Rural area	1178 (65.34)	4794 (64.27)	3884 (66.62)	
Ethic, n(%)^a^				0.0602
Han	1696 (95.71)	6923 (95.14)	5367 (95.82)	
Others	76 (4.29)	354 (4.86)	234 (4.18)	
Marriage status, n(%)^a^				<0.0001
Married	1276 (70.81)	4898 (65.78)	4587 (78.71)	
Others	526 (29.19)	2548 (34.22)	1241 (21.29)	
Occupation, n(%)^a^				<0.0001
Farmer or manual	1451 (80.97)	5805 (78.37)	4775 (82.41)	
Clerical	198 (11.05)	821 (11.08)	520 (8.97)	
Professional	65 (3.63)	469 (6.33)	222 (3.83)	
Others	78 (4.35)	312 (4.21)	277 (4.78)	
Education, n(%)^a^				<0.0001
Illiterate	1253 (70.31)	4774 (64.34)	4140 (71.40)	
Primary school	404 (22.67)	1929 (26.00)	1317 (22.71)	
Middle school or above	125 (7.01)	717 (9.66)	341 (5.88)	
Access to medical service, n(%)^a^				0.0059
Yes	1531 (84.91)	6807 (91.28)	5412 (92.86)	
No	272 (15.09)	650 (8.72)	416 (7.14)	
Smoking status, n(%)^a^				0.0019
Never	1279 (70.98)	5169 (69.38)	4116 (70.70)	
Ever smoker	222 (12.32)	882 (11.84)	778 (13.36)	
Current smoker	301 (16.70)	1399 (18.78)	928 (15.94)	
Drinking status, n(%)^a^				0.0019
Never	1323 (73.46)	5373 (72.18)	4154 (71.42)	
Ever drinker	188 (10.44)	659 (8.85)	606 (10.42)	
Current drinker	290 (16.10)	1412 (18.97)	1056 (18.16)	
Exercise status, n(%)^a^				<0.0001
Never	1216 (67.52)	5120 (69.02)	4148 (71.36)	
Ever exerciser	182 (10.11)	421 (5.68)	461 (7.93)	
Current exerciser	403 (22.38)	1877 (25.30)	1204 (20.71)	
ADL score, n(%)^a^				<0.0001
6	1350 (75.33)	6005 (80.77)	3838 (66.01)	
5	143 (7.98)	587 (7.90)	752 (12.93)	
3-4	103 (5.75)	350 (4.71)	493 (8.48)	
0-2	196 (10.94)	493 (6.63)	731 (12.57)	
Physical performance score, n(%)^a^				<0.0001
5	739 (41.42)	3831 (51.67)	2143 (36.98)	
2.5-4.5	797 (44.67)	2931 (39.53)	2787 (48.09)	
0-2.5	248 (13.90)	652 (8.79)	865 (14.93)	
MMSE score, n(%)^a^				<0.0001
24-30	764 (46.59)	3832 (56.12)	2078 (39.65)	
18-23	281 (17.13)	1146 (16.78)	968 (18.47)	
0-17	595 (36.28)	1850 (27.09)	2195 (41.88)	
Food diversity score, n(%)^a^				<0.0001
6-8	547 (30.36)	3036 (40.77)	2266 (38.92)	
4-5	618 (34.30)	2500 (33.57)	2149 (36.91)	
0-3	637 (35.35)	1911 (25.66)	1407 (24.17)	
Social activity score, n(%)^a^				<0.0001
5-8	111 (6.16)	766 (10.27)	287 (4.92)	
3-4	510 (28.29)	2602 (34.90)	1488 (25.53)	
0-2	1182 (65.56)	4088 (54.83)	4054 (69.55)	
Chronic disease score, n(%)^a^				<0.0001
0	828 (50.00)	3932 (57.38)	3518 (63.34)	
1-2	664 (40.10)	2321 (33.87)	1633 (29.40)	
≥3	164 (9.90)	600 (8.76)	403 (7.26)	

*Abbreviations*: *ADL *activities of daily living, *IQR *interquartile range, *MMSE *Mini-Mental State Examination, *SD* standard deviation

^a ^Contains missing values. Ethic: 442, marriage status:16, occupation: 99, access to medical service: 4,  education:92, smoking status: 18, drinking status: 31, exercise status: 60, ADL score: 51, physical performance score: 99, MMSE score: 1,383, food diversity score: 21, social activity score: 4, chronic disease score: 1,029

*P* values were derived from the ANOVA for continuous variables or the Chi-square test for categorical variables

### Risk of all-cause mortality with different sleep duration

In the whole population, consistent results were observed from model 1, model 2 to model 3 (Table 2). Compared to the participants with moderate sleep duration, short sleep duration was insignificantly associated with increased all-cause mortality risk (HR = 1.02, 95% CI 0.96–1.09), however, long sleep duration was positively associated with risk of all-cause mortality (HR = 1.13, 1.09–1.18).

**Table 2:** Risk of all-cause mortality among the elderly adults with different sleep duration, a cohort study in China, 2005-2019

Population	Groups	Cases/Person-years	HR (95% CI)			*P* for interaction
			Model 1	Model 2	Model 3	
Whole population	Short (≤ 6h)	1340/9518	1.08 (1.01-1.14)	1.07 (1.00-1.13)	1.02 (0.96-1.09)	
	Moderate (7-8h)	4578/35942	Ref.	Ref.	Ref.	
	Long (> 8h)	4806/22567	1.20 (1.15-1.25)	1.17 (1.12-1.22)	1.13 (1.09-1.18)	
Male	Short (≤ 6h)	485/3642	1.13 (1.02-1.25)	1.09 (0.99-1.21)	1.02 (0.92-1.13)	
	Moderate (7-8h)	1867/16294	Ref.	Ref.	Ref.	0.0300
	Long (> 8h)	1755/9759	1.14 (1.07-1.22)	1.11 (1.04-1.19)	1.08 (1.01-1.15)	
Female	Short (≤ 6h)	855/5876	1.05 (0.97-1.14)	1.06 (0.98-1.14)	1.02 (0.94-1.10)	
	Moderate (7-8h)	2711/19648	Ref.	Ref.	Ref.	
	Long (> 8h)	3051/12808	1.24 (1.18-1.31)	1.20 (1.14-1.26)	1.17 (1.11-1.23)	
Age at baseline, <80 years	Short (≤ 6h)	132/3774	1.13 (0.93-1.37)	1.07 (0.88-1.30)	1.04 (0.85-1.26)	
	Moderate (7-8h)	452/15585	Ref.	Ref.	Ref.	0.0034
	Long (> 8h)	221/5518	1.29 (1.10-1.52)	1.25 (1.06-1.47)	1.20 (1.02-1.41)	
Age at baseline, 80-89 years	Short (≤ 6h)	371/2867	1.02 (0.90-1.14)	1.01 (0.90-1.13)	0.95 (0.85-1.07)	
	Moderate (7-8h)	1155/9203	Ref.	Ref.	Ref.	
	Long (> 8h)	949/6020	1.17 (1.07-1.28)	1.12 (1.03-1.23)	1.10 (1.00-1.20)	
Age at baseline, 90-99 years	Short (≤ 6h)	458/1827	1.11 (1.00-1.23)	1.10 (0.99-1.22)	1.04 (0.94-1.16)	
	Moderate (7-8h)	1631/7085	Ref.	Ref.	Ref.	
	Long (> 8h)	1775/6265	1.21 (1.13-1.30)	1.17 (1.10-1.26)	1.12 (1.05-1.20)	
Age at baseline, ≥100 years	Short (≤ 6h)	379/1049	1.10 (0.98-1.23)	1.11 (0.98-1.24)	1.06 (0.95-1.20)	
	Moderate (7-8h)	1340/4069	Ref.	Ref.	Ref.	
	Long (> 8h)	1861/4763	1.19 (1.11-1.28)	1.16 (1.08-1.25)	1.14 (1.06-1.23)	

Model 1: adjusted for age and sex; Model 2: adjusted for age, sex, enrollment year, province, residence, education, ethic, marriage status, occupation, access to medical service, smoking status, drinking status, and exercise status; Model 3: model 2 + further adjusted for ADL score, physical performance score, MMSE score, food diversity score, social activity score, and chronic disease score.

*Abbreviations*: *CIs *confident intervals, *HR *hazard ratio

Similar pattern was observed in stratified analysis based on age and sex. The interaction effects of age, sex and sleep duration were both statistically significant (*P* < 0.05). In age-stratified analysis, the risk of long sleep duration with all-cause mortality in the subgroup of less than 80 years (HR = 1.20, 1.02–1.41) was higher than that in other age groups [for 80–90 years: 0.95 (1.00-1.20); for 90–100 years: 1.12 (1.05–1.20); and for > 100 years: 1.14(1.06–1.23)].

### The dose-response association between sleep duration and all-cause mortality

In the dose-response analysis where 25th, 50th, and 75th percentiles of its distribution were selected as the knots, we observed a J-shaped pattern, however the non-linear test was not significant (*P*_for non−linear test_ = 0.1845). Similar patterns were also observed when other knot pets were used to construct the restricted cubic spline model (Fig. 2).

## Discussion

In this prospective cohort study with the elderly participants aged 65 years and above, we found that long sleep duration was associated with an increased risk of all-cause mortality and a J-shaped pattern between them was suggestive as well. The association was robust in subgroup analyses based on age and gender.

The relationship between sleep duration and all-cause mortality in the elderly has been previously explored, results however were inconsistent. Although most studies reported a U-shaped pattern, the age distribution of the enrolled population and the definition of sleep duration were not identical [5, 17, 18]. In the First National Health and Nutrition Examination Survey with participants aged 60–86 years [17], compared to subjects with sleep duration of 7 hours, subjects with short (≤ 5 hours) and long sleep duration (≥ 9 hours) had an increased risk of mortality. In Singapore Chinese Health Study with participants aged 45–74 years, persistently short sleep (≤ 5 hours) and long sleep (≥ 9 hours) were associated with an increased risk of all-cause mortality as well when compared to the recommended sleep duration of 7 hours [18]. Åkerstedt et al. however only observed positive association among subjects less than 65 years, but not among subjects aged more than 65 years [11]. In contrast, Gangwisch et al. observed a U-shaped pattern among subjects from 60 to 86 years but not among those from 32 to 59 years [17].

Unlike the results from the previous studies [5, 17, 18], the present study suggested a J-shaped pattern between sleep duration and all-cause mortality. Our enrolled population (mean age: 89.26 years) were much older than that in the previous studies, which may partially explain the inconsistent findings, since the proportion of participants with short sleep duration tended to be decreasing with aging and the sample size in the group of short sleep duration thus tended to be smaller, which then may lead to insufficient statistical power. In addition, various definition of short sleep duration may be another explanation. For example, studies that using ≤ 5 hours as the short sleep duration reported positive associations [5, 17], while one study that using less than 6 hours did not observed a positive association [19].

In the stratified analysis by age, J-shaped pattern was consistently observed across four age groups. In previous studies [5, 17], 60 or 65 years was usually used as the cut-points because the enrolled participants were relatively younger. Consistent with the findings among the younger elderly [5, 17], our present study also found long sleep duration was associated with an increased risk of all-cause mortality among the oldest-old adults, which provided new evidence for the influence of sleep duration on those rarely investigated population. Moreover, the risk of all-cause mortality was higher in subjects aged less than 80 years than that among other age groups, which might be partially explained by survival bias: the longer the elderly live, the healthier they might be. For example, in present study, the prevalence of chronic disease was decreasing with aging, which was 50.29, 45.57, 37.07 and 34.75% in the group aged less than 80 years, 89–90 years, 90–100 years, and over 100 years, respectively.

The associations of sleep duration with all-cause mortality across gender have been inconsistent as well. In the Korean Multi-center Cancer Cohort Study, the U-shape relationship was only seen in female [5], while in Hublin et al. study, same relationship was only observed in male [7]. However, in a study with community-living adults aged more than 65 years, long sleep duration (≥ 10 hours) was associated with higher risk of mortality in both female and male [20]. Similar to our result, one population-based cohort study from China observed a J-shaped association of sleep duration with all-cause mortality risk both in male aged 40–75 years and female aged 44–79 years [19].

Although the mechanisms for the relationship between sleep duration and all-cause mortality risk remain unclear, several possible explanations have been proposed: (a) sleep fragmentation and snoring linked to long sleep duration were independent risk factor for cardio- and cerebro-vascular diseases [21, 22], which are the leading causes of mortality worldwide; (b) long sleep duration may lead to lethargy and fatigue, decrease the resistance to stress and disease, and then increase the consequent mortality risk [23]; (c) long sleep duration demonstrated strong associations with chronic diseases including cognitive impairment, depression, mental distress, stroke, coronary heart disease, and diabetes [6, 24–26], which may mediate the relationship between long sleep duration and mortality, although health conditions were adjusted for in present and previous studies; (d) photoperiod reduction arising from long sleep duration may increase the risk of mortality directly [27]. Similar to long sleep duration, the influence of short sleep duration on mortality may be mediated by some disorders or diseases, such as obesity, cancer, infectious and chronic diseases [24, 28, 29].

Strengths of our study include the prospective cohort study design, long follow-up time, and a large sample size. In addition, taking into account the change of sleep duration in the follow-up enabled us to more precisely evaluate the association, which may eliminate the misclassification of exposure status to a large extent. Moreover, the studied population was mainly consisted of oldest-old adults, such as octogenarians, nonagenarians, and centenarians, for whom the influence of sleep duration on all-cause mortality was rarely explored.

This study also has several limitations. Firstly, sleep duration, like many previous studies, was collected through self-report, which might lead to misclassification of exposure. However, studies suggested that self-reported sleep duration was well-correlated with sleep duration objectively measured by actigraphy- and single-night in-home polysomnography [30, 31]. Secondly, although a wide range of lifestyle factors and health conditions (i.e., cognitive impairment, social activity engagement, chronic disease status) were adjusted for in our study, the influence of residual confounding, caused by measured or unmeasured confounders (e.g., the severity of chronic diseases), cannot be ruled out. However, the consistent results from main analyses to subgroup analyses argue against that residual confounding would overturn our results in this case. Thirdly, the study population represented the elderly individuals in China, therefore the generalization of present findings to other age groups, areas or ethnicities should be done with caution.

## Conclusions

In summary, we found that long sleep duration was associated with a higher risk of all-cause mortality among the elderly population. Given sleep duration can be easily measured in practice, it should be regarded as a useful signal to alert healthcare providers, family members, and the elderly population for the risk of mortality.

## Data Availability

All data used in this study was stored at Peking university (http://opendata.pku.edu.cn/), researchers can contact qwang@nsd.pku.edu.cn to apply for the data.

## Abbreviations

CLHLS: Chinese Longitudinal Healthy Longevity Survey
HRs: Hazard ratios
CIs: Confidence intervals
SD: Standard deviation
ADL: Activities in daily living
MMSE: Mini-Mental State Examination
